# The oxidative stress adaptor p66Shc is required for permanent embryo arrest in vitro

**DOI:** 10.1186/1471-213X-7-132

**Published:** 2007-11-29

**Authors:** Laura A Favetta, Pavneesh Madan, Gabriela F Mastromonaco, Elizabeth J St John, W Allan King, Dean H Betts

**Affiliations:** 1 Department of Biomedical Sciences, Ontario Veterinary College, University of Guelph, Guelph, Ontario N1G 2W1, Canada

## Abstract

**Background:**

Excessive developmental failure occurs during the first week of *in vitro *embryo development due to elevated levels of cell death and arrest. We hypothesize that permanently arrested embryos enter a stress-induced "senescence-like" state that is dependent on the oxidative stress-adaptor and lifespan determinant protein p66Shc. The aim of this study was to selectively diminish p66Shc gene expression in bovine oocytes and embryos using post-transcriptional gene silencing by RNA-mediated interference to study the effects of p66Shc knockdown on *in vitro *fertilized bovine embryos.

**Results:**

Approximately 12,000–24,000 short hairpin (sh)RNAi molecules specific for p66Shc were microinjected into bovine germinal vesicle stage oocytes or zygotes. Experiments were comprised of a control group undergoing IVF alone and two groups microinjected with and without p66Shc shRNAi molecules prior to IVF. The amount of p66Shc mRNA quantified by Real Time PCR was significantly (P < 0.001) lowered upon p66Shc shRNAi microinjection. This reduction was selective for p66Shc mRNA, as both histone H2a and p53 mRNA levels were not altered. The relative signal strength of p66Shc immuno-fluorescence revealed a significant reduction in the number of pixels for p66Shc shRNAi microinjected groups compared to controls (P < 0.05). A significant decrease (P < 0.001) in the incidence of arrested embryos upon p66Shc shRNAi microinjection was detected compared to IVF and microinjected controls along with significant reductions (P < 0.001) in both cleavage divisions and blastocyst development. No significant differences in p66Shc mRNA levels (P = 0.314) were observed among the three groups at the blastocyst stage.

**Conclusion:**

These results show that p66Shc is involved in the regulation of embryo development specifically in mediating early cleavage arrest and facilitating development to the blastocyst stage for in vitro produced bovine embryos.

## Background

**Figure 1:**
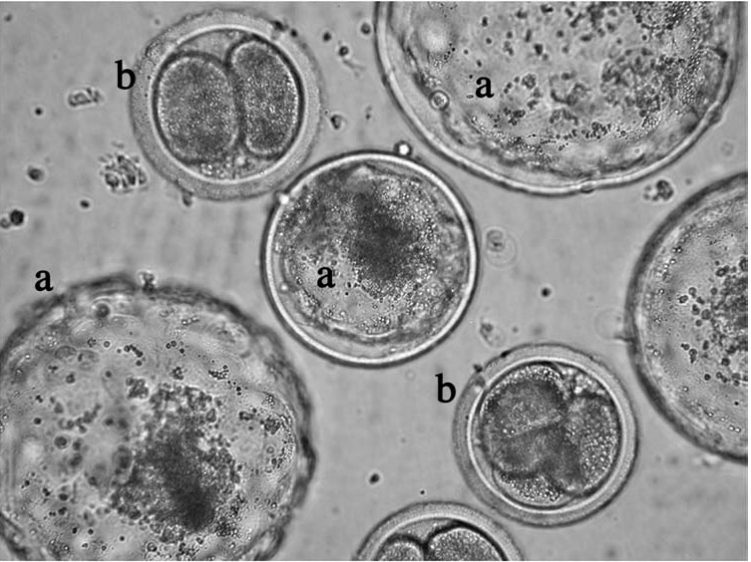
Appearance of 2–4 cell arrested embryos at day-8 post insemination. Morphological appearance of day-8 *in vitro *produced bovine embryos. Embryos at this time after *in vitro *fertilization usually reached the blastocyst stage (a), while arrested embryos still appeared as morphologically normal 2–4 cell embryos (b). (Magnification: 400×).

One feature of *in vitro *produced mammalian embryos is the high frequency of early developmental failure brought on by sub-optimal culture environments [1-3]. Fewer than half of all *in vitro *fertilized (IVF) bovine embryos reach the blastocyst stage of development [4] with many of these unable to sustain development following embryo transfer [5]. The reasons for this high rate of *in vitro *embryo demise remains unclear, but it has been proposed as a protective mechanism for preventing further development of abnormal, poor quality embryos. Almost half of all arrested human embryos display chromosomal abnormalities [6], and significantly more chromosomal aberrations are observed, alongside delayed development, for *in vitro *produced bovine embryos compared to their *in vivo*-derived counterparts [7,8]. Blastomeres with characteristic features of apoptosis, such as cytoplasmic, nuclear and DNA fragmentation, have been detected in both *in vitro *and *in vivo *derived embryos, indicating that high levels of apoptosis might play a role in early embryo death [9,10]. *In situ *TUNEL labelling has identified a greater incidence of apoptotic nuclei in cultured bovine blastocysts compared to those derived *in vivo *[11]. Interestingly, we [1,12,13] and others [11,14] have observed no morphological or biochemical signs of apoptosis during the early 2–4 cell cleavage stages of bovine embryogenesis. It is at this early developmental stage that approximately 14% of all *in vitro *produced (IVP) bovine embryos permanently arrest in a senescence-like state (Fig. 1) [2,15].

Cellular or replicative senescence is the well known *in vitro *phenomenon whereby most proliferating somatic cell types irreversibly cease in their replicative capacity after a fixed number of population doublings and display distinct morphological features of "aged" cells [16,17]. Senescence is triggered by a p53-dependent signalling cascade associated with the shortening of telomeres, the tandem G-rich repetitive, non-coding DNA sequences and specific binding proteins at the ends of linear chromosomes [18-21]. We have previously shown that permanent cell growth arrest of cultured bovine somatic cells is associated with telomere shortening [22], increased levels of serine 20-p53 phosphorylation, and elevated levels of oxidative damage [23]. Since permanent growth arrest/cellular senescence occurs prematurely under conditions of elevated oxidative stress [24,25] and can be activated by disruption of the telomere structure itself, even at maximally long telomere lengths [26,27], we hypothesize that arrested mammalian embryos permanently arrest in their development by a similar stress signalling pathway.

At the cellular level, an oxidative stress sensor that can provide a key understanding of cell senescence and permanent embryo arrest is p66Shc, the recently identified protein belonging to the Shc family of adaptors for signal transduction in mitogenic and apoptotic responses. p66Shc is involved in signal transduction pathways that regulate the cellular response to oxidative stress and life span and has been shown to act as a downstream target of p53-mediated elevation of intracellular oxidants, cytochrome c release and apoptosis [28,29]. p66Shc is important in the signalling response to ROS and propagation of apoptotic signals in mouse fibroblasts [30,31]. Remarkably, p66Shc(-/-) mice exhibit increased resistance to oxidative stress and have a ~30% longer lifespan than wild-type control animals with no apparent side effects [32]. We have measured elevated levels of p66Shc mRNA and protein in senescent somatic cells and in permanently arrested 2–4 cell bovine embryos [2,23]. Recently, we have demonstrated that p66Shc is significantly more abundant in an embryo population that exhibits higher frequencies of embryo arrest and quantities of intracellular ROS [15]. Taken together, these data suggests that p66Shc regulates an oxidative stress-induced senescence pathway that triggers permanent embryo arrest. The objective of this study was to elucidate a functional role for p66Shc in embryo arrest by selectively decreasing p66Shc levels in bovine oocytes and embryos using the post-transcriptional gene silencing technique of RNA interference (RNAi).

## Results

### Quantification of p66Shc, H2a and p53 mRNA transcripts

Determination of the number of p66Shc, H2a and p53 mRNA transcripts was carried out by Real Time PCR in arrested 2–4 cell embryos collected at day 8 post-insemination, in early cleaving embryos (28 hours post insemination (hpi)) that show very low percentage of embryo arrest and in late cleaving embryos (29 to 48 hpi) that are more likely to arrest, in accordance to previous observations [2]. These data are summarized in Table 1 along with the amounts of these specific gene transcripts in day 8 blastocysts. Significantly greater quantities of p66Shc mRNA transcripts (2.3–3.4 fold greater) were measured in both arrested (P < 0.0001) and late cleaving embryos (P < 0.005) that are more likely to arrest compared to early cleaving 28 hpi embryos. No differences in H2a or p53 mRNA levels were detected among early and late cleaving embryos, arrested embryos, and blastocysts. The numbers of p66Shc mRNA transcripts at the blastocyst stage were significantly (P < 0.001) lowered compared to those measured in early cleaving and arrested embryos (Table 1).

**Table 1:** Quantity of p66Shc, p53 and H2a mRNA transcripts in early cleaving, late cleaving, 2–4 cell arrested embryos and bovine blastocysts

	Quantity of mRNA transcripts/embryo (pg)			
	
	Early cleaving embryos (28 hpi)	Late cleaving embryos (29 to 48 hpi)	2–4 cell arrested embryos	Blastocysts
p66Shc	1.20 ± 0.30 × 10^-5 a^	2.80 ± 0.32 × 10^-5 b^	4.02 ± 0.21 × 10^-5 c^	0.50 ± 0.07 × 10^-5 d^
H2a	0.53 ± 0.09 × 10^-3 a'^	0.43 ± 0.12 × 10^-3 a'^	0.46 ± 0.13 × 10^-3 a'^	0.43 ± 0.13 × 10^-3 a'^
p53	0.95 ± 0.13 × 10^-6 a"^	1.01 ± 0.15 × 10^-6 a"^	0.81 ± 0.22 × 10^-6 a"^	1.70 ± 0.50 × 10^-6 a"^

Values represent mean ± SEM

Different superscripts differ significantly (^a,b^P < 0.005; ^a,c^P < 0.0001; ^a,d^P < 0.001)

### Determination of the number of p66Shc shRNAi molecules to microinject

In the attempt to down-regulate p66Shc mRNA expression, we wanted to microinject the amount of p66Shc hairpin RNAi molecules equivalent to the number of copies of p66Shc transcripts measured in arrested 2–4 cell embryos. The average amount of p66Shc mRNA transcripts in arrested 2–4 cell embryos, as detected by Real Time PCR, was 4.02 ± 0.1 × 10^-5 ^pg/embryo (Table 1). Using the size of the amplified PCR fragment (336 bp) and the molecular weight of the nucleotides, the quantity (pg) of p66Shc mRNA in arrested 2–4 cell embryos were transformed to equivalent numbers of p66Shc mRNA transcripts of approximately 11,800 ± 600. Therefore, approximately 12,000 p66Shc short hairpin RNAi molecules were microinjected in the subsequent experiments. To further verify a dose-dependent silencing/reduction of p66Shc protein levels we also injected twice (~24,000 molecules) the amount of p66Shc shRNAi molecules.

### Successful selective down-regulation of p66Shc

**Figure 2:**
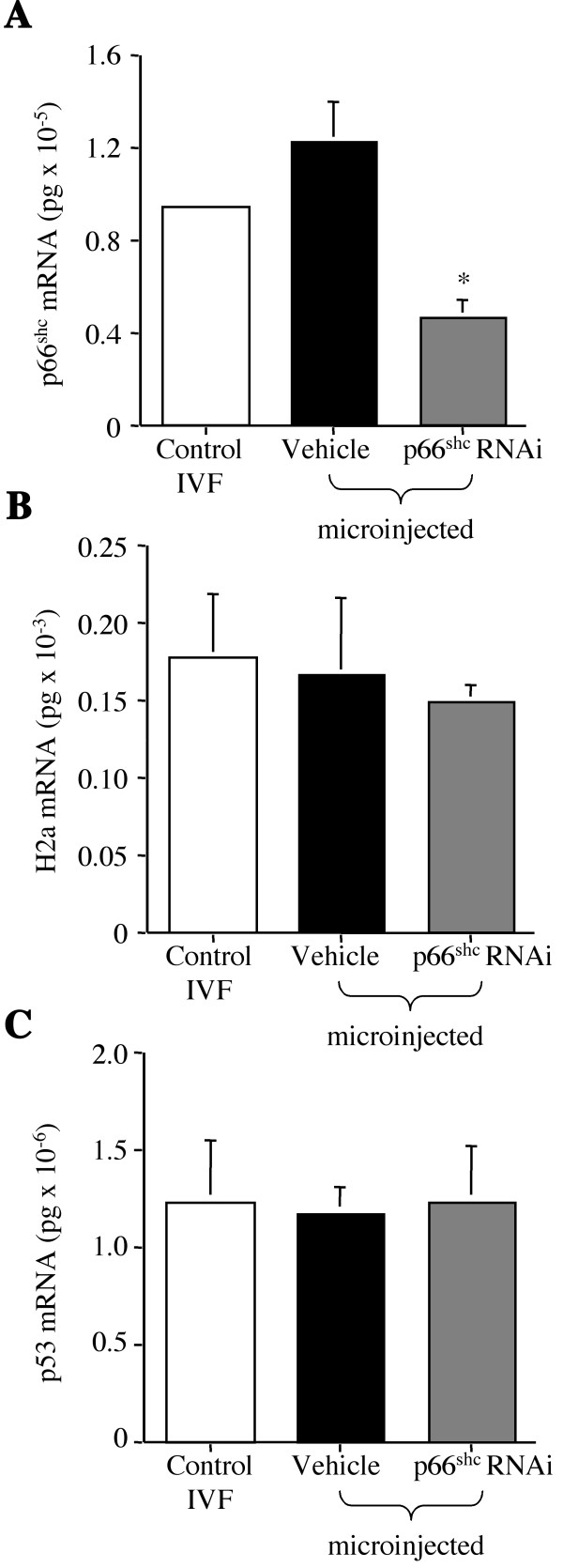
Real Time PCR quantification of p66Shc mRNA levels at the 2-cell stage following RNAi. Real Time PCR analysis of p66Shc (A) and H2a (B) mRNA levels in 35 hpi embryos following control IVF, microinjection of the vehicle and microinjection with 12,000 p66Shc hairpin RNAi molecules. p66Shc mRNA levels decreased in p66Shc RNAi microinjected embryos (*P < 0.001), while Histone H2a mRNA levels showed no significant differences (P = 0.744) among the three groups. This result provided evidence that the down-regulation of p66Shc mRNA levels following p66Shc hairpin RNAi molecule injection is selective for p66Shc transcripts. Real Time PCR analysis of p53 mRNA levels (C) for the three groups was also performed, showing no significant differences (P = 0.98). The experiments were conducted on three different pools of 80–100 embryos (n = 3) and replicated three times (r = 3) for each sample. The data were normalized for the content in one embryo.

To assess whether p66Shc expression was selectively down-regulated by the injection of approximately 12,000 and 24,000 p66Shc short hairpin RNAi molecules, we quantified p66Shc mRNA and protein levels in 35 hpi embryos by Real Time PCR and by quantitative immunoflorescence, respectively. The IVF control group contained 0.92 ± 0.02 × 10^-5 ^pg of p66Shc mRNA, the vehicle alone microinjected group 1.2 ± 0.2 × 10^-5 ^and the p66Shc shRNAi microinjected group 0.46 ± 0.06 × 10^-5 ^pg of p66Shc transcripts (Fig. 2A). Therefore, this result demonstrated that microinjection of oocytes at the GV stage with the p66Shc shRNAi molecule resulted in significantly lower levels of p66Shc mRNA (P ≤ 0.001) by 54% attained in comparison to the control and vehicle microinjected groups. To confirm that the down-regulation was selective towards p66Shc mRNA, p66Shc shRNAi treatment did not significantly affect (P = 0.744) the mRNA levels of the housekeeping gene histone H2a in the three groups considered (Fig. 2B). In addition, Real Time PCR evaluation of the p53 mRNA levels following p66Shc down-regulation was also performed obtaining no significant differences (P = 0.98) in p53 mRNA transcript abundance among the control, vehicle microinjected and p66Shc shRNAi molecule microinjected groups (Fig. 2C).

**Figure 3:**
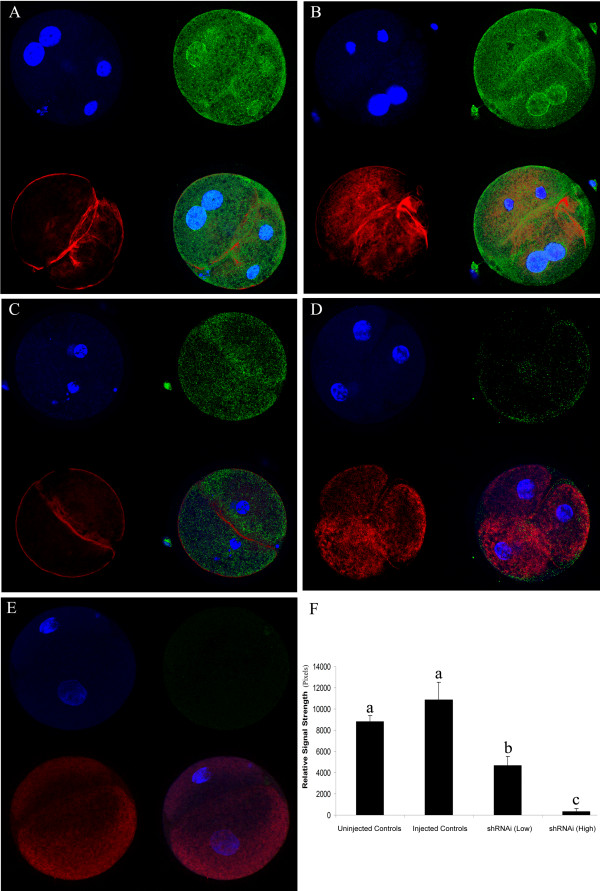
p66Shc protein levels following microinjection of p66 shRNA interfering molecules. The distribution of p66Shc in early bovine embryos was assessed (A, B, C and D). Green, red and blue colors in each representative photomicrograph indicate positive staining for the respective primary antibody: p66Shc (FITC), F-actin (Rhodamine phalloidin), and nuclei (DAPI) respectively. Panels A and B represent Uninjected and Injected (Vehicle) controls respectively. Panel C (shRNAi low dose = ~12,000 molecules) and Panel D (shRNA high dose = ~24,000 molecules) display a marked reduction in p66 fluorescence. Panel E represent the no primary antibody controls. Scion Image analysis resulted in the quantification of FITC immunofluorescence (p66Shc) across different groups (F). Relative signal strengths are presented as the mean ± S.E.M. representative of three independent replicates. Bars with different letters represent significant differences in relative signal strength between treatment groups (*P *≤ 0.05).

The p66Shc protein levels and distribution patterns in non-injected and vehicle microinjected groups were not different, displaying relatively high immunofluorescence intensities and punctate staining patterns in the cytoplasm and nuclei of all embryos analyzed (Fig. 3A,B). Unexpectedly, a peri-nuclear ring of staining around the nuclei was readily apparent (Fig. 3A,B). In contrast, 35 hpi embryos that had been injected with ~12,000 (Fig. 3C) and ~24,000 (Fig. 3D) p66Shc shRNAi molecules showed visible reductions in p66Shc fluorescence staining intensities. Control embryos treated with the secondary FITC-conjugated antibody alone (no primary antisera) exhibited virtually no staining (Fig. 3E). Quantitative analysis of the relative signal strength of p66Shc fluorescence revealed a significant (P < 0.05) reduction in pixel numbers for both low (12,000) and high (~24,000) p66 shRNAi microinjected groups compared to non-injected and vehicle injected controls (Fig. 3F). The knockdown efficiency of p66Shc protein was 52.5% for 12,000 shRNAi molecules and 96.8% for 24,000 shRNAi molecules compared to p66Shc levels in vehicle-injected embryos.

### Effects of p66Shc knockdown on in vitro embryo development

**Figure 4:**
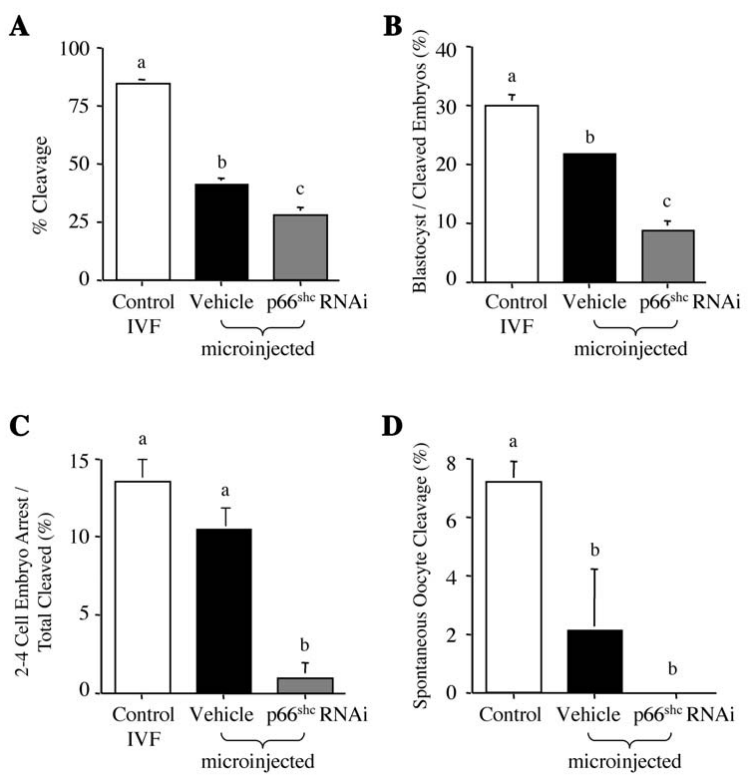
Assessment of cleavage, blastocyst, 2–4 cell embryo arrest and spontaneous embryo cleavage rates following microinjection with p66Shc hairpin RNAi molecules. Cleavage rate (A) and spontaneous cleavage rate (D) were evaluated at day-2 post insemination, while blastocyst rate (B) and arrest rate (C) were evaluated at day 8–9 post insemination. Although cleavage and blastocyst frequencies were significantly decreased (*P *< 0.001) in the embryos microinjected with the vehicle alone, an even greater significant decrease (*P *< 0.001) was observed in the p66Shc shRNAi microinjected oocytes (A, B). We observed a significant decrease (*P *< 0.05) in spontaneous oocyte cleavage rate following microinjection (D). A significant decrease in the frequency of arrested 2–4 cell embryos was achieved only following microinjection of short hairpin RNAi molecules against p66Shc (C). Bars with different letters represent significant differences between groups (*P *≤ 0.05).

The percentage of embryo cleavage varied significantly among the three groups with 84.1 ± 1.9% of oocytes cleaved after fertilization in the control non-injected group, 41.0 ± 2.4% in the group microinjected with only the vehicle and 27.7 ± 3.2% when the p66Shc hairpin RNAi molecules were microinjected (P < 0.001) (Fig. 4A). The developmental frequencies to the blastocyst stage, calculated as the number of blastocysts over the total number of cleaved oocytes, was 29.8 ± 1.6%, 21.8 ± 0.5% and 8.7 ± 1.4% for the control, vehicle and p66Shc RNAi microinjected groups respectively (P < 0.001) (Fig. 4B). Subsequent injections of scrambled short-interfering (si)RNA control molecules, non-specific for any endogenous transcripts, had no affect on embryo arrest frequencies and developmental potential of bovine embryos compared to vehicle-injected embryos (data not shown). Interestingly, while there were no significant differences in the percentage of arrested 2–4 cell embryos between the control (13.3 ± 0.8%) and the group microinjected with the vehicle (10.8 ± 0.7%), there was a significant decrease (P < 0.001) in the percentage of permanent embryo arrest in the group where p66Shc was down-regulated (Fig. 4C), which showed only 0.9 ± 0.9% of cleaved embryos arresting. Assessment of spontaneous oocyte cleavage revealed 7.2 ± 0.7% oocytes spontaneously cleaved in the control group, a significant decrease (P < 0.05) in spontaneous cleaved oocytes after microinjection with 2.1 ± 2.1% in the vehicle microinjected group, and no spontaneous oocyte cleavage in the p66Shc microinjected group. The differences in spontaneous oocyte cleavage between the vehicle and p66Shc shRNAi injected groups were not significant (P > 0.05) (Fig. 4D).

### No differences in p66^Shc ^transcript levels were detected in embryos that reached the blastocyst stage

**Figure 5:**
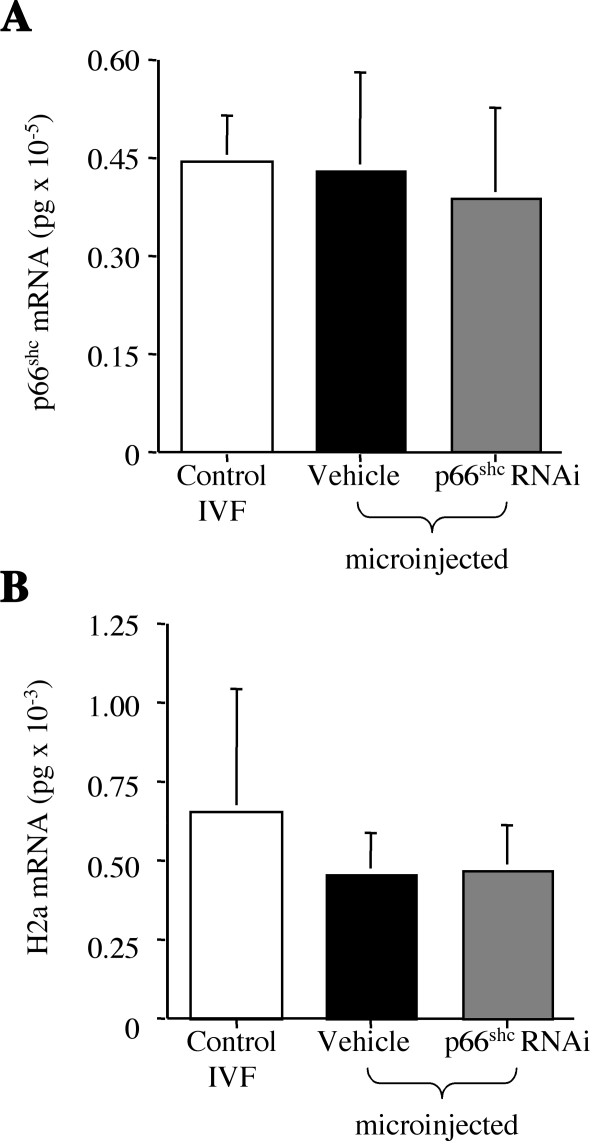
Real Time PCR quantification of p66Shc mRNA levels at the blastocyst stage following RNAi. Real Time PCR analysis of p66Shc (A) and H2a (B) mRNA levels in day 8–9 blastocysts obtained following control IVF, microinjection of the vehicle and microinjection of 12,000 p66Shc short hairpin shRNAi molecules. No significant differences (*P *= 0.314) in the p66Shc mRNA levels were measured (A). The quantification of the histone H2a mRNA levels was used as a control, observing no significant differences (*P *= 0.312) (B). The experiments were conducted on three different pools of 80–100 embryos (n = 3) and replicated three times (r = 3) for each sample. The data were normalized for the content in one blastocyst.

Quantification by Real Time PCR of p66Shc mRNA in blastocysts collected at day-9 post-insemination from the three groups, showed no significant differences in p66Shc mRNA levels (p = 0.314) among the different groups (Fig. 5A), with 0.59 ± 0.1 × 10^-5 ^pg of p66Shc mRNA in the control group, 0.57 ± 0.2 × 10^-5 ^pg in the vehicle microinjected group and 0.51 ± 0.2 × 10^-5 ^pg in the p66Shc shRNAi microinjected group. The amount of p66Shc transcripts in each group reflects the amount normally detected at the blastocyst stage (Table 1). Quantification of the housekeeping gene, H2a, was used as a control and showed no significant differences (p = 0.312) (Fig. 5B).

## Discussion

The results presented here demonstrate the successful application of RNA interference (RNAi) in selectively diminishing the oxidative stress adaptor protein p66Shc during *in vitro *bovine embryo development. Based on our previous findings that showed significantly elevated levels of p66Shc and reactive oxygen species in permanently arrested 2–4 cell bovine embryos [2,15], the goal in this study was to decrease p66Shc levels in developing bovine oocytes and embryos to elucidate a functional role for p66Shc in early embryo arrest. We significantly decreased the number of p66Shc mRNA transcripts and protein by approximately 54% by microinjecting ~12,000 short hairpin (sh)RNAi molecules specific for the bovine p66Shc mRNA sequence into GV stage oocytes. Concomitant with this reduction in p66Shc mRNA and protein levels, we observed a significant reduction in permanently arrested 2–4 cell embryos of approximately 93%. Therefore, this study clearly demonstrates a direct functional association between high levels of p66Shc with early embryo arrest. However, down-regulation of p66Shc also induced a significant reduction in the number of cleaved embryos and blastocysts signifying that it may play a vital role in facilitating early embryonic development as well.

**Figure 6:**
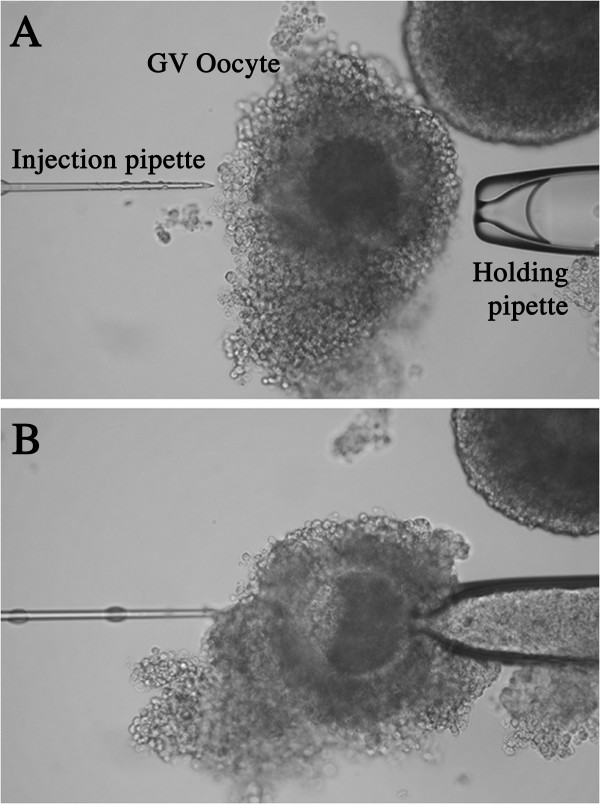
Oocyte microinjection. The short hairpin (sh)RNAi microinjection of a the germinal vesicle (GV) stage bovine oocyte. (A) The oocyte has been localized and is subsequently microinjected with shRNAi molecules (B). The oocyte is being held by the holding pipette and microinjected by a pipette of 4 μm in diameter and beveled at 35°. Care was taken not to strip off the cumulus cells surrounding the GV oocytes and to avoid perforating the germinal vesicle during microinjection.

Since permanent embryo arrest occurs before the major activation of the embryonic genome [33-35], microinjection of the p66Shc hairpin RNAi molecules was required at the germinal vesicle (GV) stage to maximize the effects of RNAi mediated reduction of endogenous p66Shc transcripts. Microinjection was carried out on cumulus-intact GV oocytes, but this presented several challenges. Accordingly, care was taken to minimize the damage to COCs by the holding pipette, which readily stripped off the cumulus cells, and to reduce oocyte injury by the injection pipette since the ooplasm contents were not easily visible and piercing of the GV was possible (Fig. 6). Denuded immature oocytes do exhibit a decreased ability to reach the second metaphase (MII stage), with a greater incidence of abnormal fertilization, reduced pronuclei formation, and reduced cleavage and blastocyst development [36-39]. When we microinjected embryos without the p66Shc shRNAi molecules (vehicle control), we found a significant decrease in these parameters with 41.0 ± 2.4% of embryos cleaved, 21.8 ± 0.5% of embryos that develop to the blastocyst stage and 10.8 ± 0.7% of embryos that arrested. It appears that the micromanipulation procedure itself affects the developmental potential of IVP bovine embryos reaching the blastocyst stage in vitro. A similar decrease in cleavage and blastocyst rates has been reported after pronuclear injection of bovine zygotes [40]. To improve oocyte survival following microinjection of RNAi molecules the use of cytochalasin B and cycloheximide treatment prior to puncture have improved oocyte survival rates to levels comparable to non-punctured control oocytes [41].

Only a limited number of studies have been carried out using RNA interference in mammalian oocytes and embryos [41-46]. Svoboda et al. (2000) were able to down-regulate the expression of specific target genes up to 80% in a dose-dependent manner. They microinjected short hairpin RNAi molecules in mouse oocytes, obtaining a viable down-regulation using a range between 1.0 × 10^4 ^and 1.5 × 10^6 ^molecules in a volume of 5 pl [47]. Wianny and Zernicka-Goetz (2000) microinjected mouse oocytes with 0.1 to 2.0 μg/μl of hairpin RNAi molecules in a larger volume, up to 10 pl, to down-regulate their genes of interest. Marked success was also achieved by microinjection of the short interfering siRNA expression vector directly into the nuclei of 2-cell mouse embryos in a concentration of 10 ng/μl [46]. In the present study, approximately 12,000 p66Shc hairpin RNAi molecules were microinjected in GV-stage bovine oocytes, representing the number of p66Shc mRNA molecules previously quantified in arrested 2-cell embryos [2]. The microinjection volume we utilized needed to be significantly smaller than what was used previously for mouse oocytes. Microinjection of a volume greater than 0.25 pl resulted in mechanical lysis of the GV bovine oocytes. This species-difference might be due to a greater fragility of bovine oocytes versus mouse oocytes or to different parameters, such as the optimal pressure utilized for the microinjection and the microinjection needle diameter.

Early cleaving co-culture embryos known to display high developmental potential and a low incidence of embryo arrest [2], exhibit an average p66Shc mRNA concentration of 1.20 ± 0.30 × 10^-5 ^pg per embryo, while late cleaving embryos, which are significantly more likely to arrest, contain a p66Shc mRNA amount of over twice as much (2.80 ± 0.32 × 10^-5 ^pg) per embryo (Table 1). The amount of p66Shc mRNA is even higher in arrested 2–4 cell embryos collected at day-8 post-insemination, at 4.02 ± 0.21 × 10^-5 ^pg per embryo (Table 1). These results, along with the consensus that the major activation of embryonic transcription occurs at the 8–16 cell stage in bovine embryos [48,49], suggest that the increased levels of p66Shc mRNA and protein in arrested embryos may be due to an increase in p66Shc mRNA stability and/or protein stability through post-transcriptional or post-translational modifications. Recently, the small GTPase rac1 has been shown to induce phosphorylation-dependent increase in the stability of p66Shc in various somatic cell lines [50]. Future experiments will elucidate whether the increase in p66Shc in arrested embryos is due to *de novo *transcription, increased transcript stability and/or protein stability.

There was no increase in spontaneously cleaved oocytes upon RNAi microinjection detected in this study, in contrast to the results obtained in the mouse [43,45]. Spontaneous oocyte cleavage induced by the microinjection procedure itself, while occurring in some species such as mouse [51], humans [52] and hamsters [53], does not readily occur in the bovine [54]. In the absence of chemical activation, approximately 4% of injected bovine oocytes cleaved [54], which is consistent with the results presented in this study where 2.1 ± 2.0% of injected oocytes spontaneously cleaved following microinjection. Most spontaneously cleaved bovine oocytes exhibit developmental delay/arrest [55], suggesting that the reduction in spontaneous cleaved oocytes in the microinjected groups compared to non-injected controls (Fig. 4D) may account for the reduction in embryo arrest. However, there was also significant reduction in spontaneous oocyte cleavage in the vehicle-microinjected group without a concomitant reduction in the proportion of embryos permanently arresting at the 2–4 cell stage. This result further supports that the decrease in arrested embryos observed for the p66Shc shRNAi microinjected group was not due to a reduction in spontaneous oocyte cleavage but to the knockdown in p66Shc levels.

P66Shc knock-out mice are viable, but the fertility of p66Shc null females has not been reported [32]. In this study, RNAi-mediated reduction in p66Shc mRNA and protein significantly reduced the proportion of embryos arrested at the 2–4 cell stage (0.9 ± 0.9%), but only 8.7 ± 1.4% developed to the blastocyst stage (Fig. 4B), suggesting that escape from arrest leads to embryo demise by other means at later developmental stages and that p66Shc has a variety of functions during early embryo development. The reduction in blastocyst yield might suggest RNAi toxicity to the oocytes/embryos, but this is not likely since histone H2a and p53 mRNA levels were unchanged for both the 2-cell embryo and blastocyst stages after p66Shc RNA interference. Any insult/toxicity would greatly affect the expression of most genes [3,56,57], rather than isolated individual loci. The use of long (>30 nt) dsRNA molecules have been shown to trigger a sequence non-specific interferon response [58], however the use of short interfering and short hairpin RNAi molecules in mammalian cells and embryos have not shown any toxicity or interferon response [41,44,58]. The lack of alterations in p53 mRNA levels also suggest that p53 is not affected by p66Shc mRNA down-regulation, consistent with our previous observations, which propose that embryo arrest and death are p53-independent events in early bovine embryos [2,12]. The levels of p66Shc transcripts after a 54% p66Shc knockdown with ~12,000 shRNAi molecules in 35 hpi, 2–4 cell embryos (0.46 ± 0.06 × 10^-5 ^pg/embryo) is equivalent to the amount of p66Shc mRNA normally observed in a developing bovine blastocyst (0.50 ± 0.07 × 10^-5 ^pg) (Table 1), suggesting that we were working in a physiological range typically measured in blastocysts. At the blastocyst stage no differences in p66Shc mRNA levels were observed between the control, vehicle microinjected and p66Shc RNAi microinjected groups (Fig. 2). This result indicates that the RNAi molecules either induced only a temporary effect on endogenous p66Shc levels, or that an optimal concentration of p66Shc is necessary for continued development.

Besides p66Shc's role in normal cell management of stress and damage repair, p66Shc functions in regulating mitochondrial metabolism [59]. In the absence of p66Shc, mouse embryonic fibroblasts and PC12 cells both exhibit a reduction in mitochondrial oxidative phosphorylation capacity, which was partially offset by increased levels of aerobic glycolysis and lactate production [59]. This altered mitochondria metabolism in p66Shc deficient cells is the most likely explanation for the reduced blastocyst frequencies observed for the p66Shc knockdown embryo group. Quantitative assessment at timed stages of development coupled with a titrated silencing of p66Shc under culture conditions with varying oxygen levels and energy sources will help clarify the metabolic role of this protein during early embryo development.

p66Shc is an adaptor protein that is activated in response to oxidative stress and appears to play a crucial role in mediating cell death and cellular senescence [23,32]. Conversely, Trinei *et al*. (2002) showed that p66Shc is an oxidative stress inducer, increasing reactive oxygen species (ROS) production [28]. It is possible that the reduction in permanently arrested 2–4 cell embryos in the p66Shc shRNAi injected group is due to a decline in mitochondrial ROS production and an increase in stress resistance. Recent studies have shown that oxidative stress-activated p66Shc can translocate into the mitochondrial intermembrane space where it interacts with reduced cytochrome c to produce H_2_O_2 _which promotes the opening of permeability transition pores allowing for the release of ROS and other constituents into the cytosol [29,60,61]. The localization of p66Shc protein throughout and as distinct foci within the cytoplasm of bovine embryos corroborates with this data. ROS are involved in the redox-dependent regulation of various cellular functions, including energy metabolism and the response to stress or growth signals [62]. Our previous research demonstrated that elevated oxygen tensions increase the levels of p66Shc, intracellular ROS and oxidative damage in bovine fibroblasts and embryos [15,23], supporting other work that shows that oxidative stress mediates p66Shc's function [28,61,63,64]. Interestingly, embryos injected with the high dose (~24,000) of p66Shc shRNAi molecules appeared to cleave faster than those injected with the low does (~12,000) of p66Shc RNAi hairpin molecules (data not shown), supporting p66Shc's other known role in antagonizing mitogenic signalling [65].

Embryo arrest and continued embryo development may therefore be regulated, in part, by the translocation of p66Shc into the inter-mitochondrial space where it controls trans-membrane potential altering mitochondrial metabolism and/or generating and releasing ROS into the cytosol causing cellular damage that triggers permanent cell cycle arrest at a developmental stage (2–4 cell embryo) that is in an anti-apoptosis state [1] or apoptosis at other developmental stages. p66Shc has been shown to regulate redox-dependent inactivation of members from the forkhead transcription factor family triggering their translocation from the nucleus to the cytosol [64]. Forkhead transcription factors have been reported to regulate the expression of anti-apoptosis genes and several antioxidant enzymes, including superoxide dismutase (SOD) and catalase [64,66,67]. The nuclear and peri-nuclear localization of p66Shc observed in this study and elsewhere [68] may account for this extra-mitochondrial role for p66Shc. RNAi-mediated knockdown of p66Shc in embryos already deemed arrested and in embryos at later developmental stages will reveal the developmental capacity of arrested embryos and may uncover the aetiology of the permanently arrest state and other p66Shc function(s) during later stages of early embryo development.

Finally, there is now supporting evidence that telomere-driven senescence is triggered by mitochondrial production of ROS [69,70]. ROS-mediated disruption of the telomere structure may explain the high rates of developmental arrest of *in vitro *produced embryos [17]. Preliminary evidence in our lab detecting phosphorylated histone γ-H2A.X foci in arrested 2–4 cell embryos but not in proliferating 2–4 cell embryos support this possibility since phosphorylation of histone H2A.X appears to facilitate the formation of DNA damage foci around uncapped telomeres [71]. Based on the immunolocalization studies it is tempting to speculate that p66Shc may be involved in a cycling cascade of events that triggers and maintains permanent embryo arrest through a ROS-mediated and response pathway that permanently maintains a telomeric DNA damage state.

## Conclusion

In conclusion, using the post-transcriptional gene silencing approach of RNA interference by microinjecting short hairpin RNAi molecules into bovine oocytes/embryos, we were able to selectively diminish the levels of p66Shc and correlated this knockdown with a significant reduction in the incidence of 2–4 cell embryo arrest and with a significant decrease in embryo development to the blastocyst stage. These apparent contradictory roles for p66Shc in both permanent embryo arrest and developmental competence implies that p66Shc functions must be tightly regulated. Escape from permanent cell cycle arrest may alleviate some cases of age-related infertility, or at least provide a less controversial source of embryonic stem cells from arrested embryos original deemed as non-viable, biological waste.

## Methods

### Plasmid preparation and cells transformation

**Figure 7:**
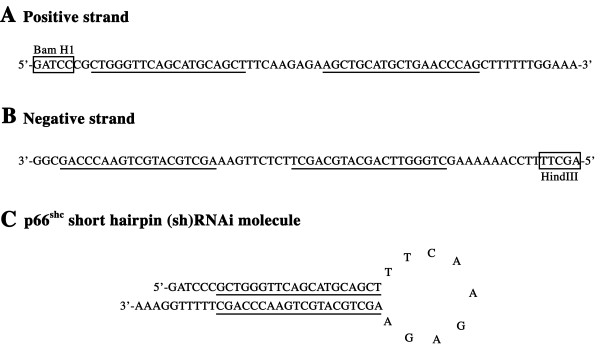
P66Shc short hairpin (sh)RNAi molecule sequence. The positive strand (A) was designed with a BamH1 site at the 5' end followed by the 19 nucleotide specific for bovine p66Shc (underlined), a loop of 9 non-complementary nucleotides and the reverse complementary sequence of the 19 nucleotides specific for bovine p66Shc (underlined). The negative strand (B) consists of the reverse complementary to the positive one, without the BamH1 site, but with a HindIII restriction site at its 5' end. (C) The represented shRNAi structure that is formed within the oocyte/embryo upon microinjection.

Two DNA oligonucleotides were synthesized at the Guelph Molecular Supercentre (Guelph, ON, Canada). A positive strand containing 19 nucleotides specific to the p66Shc mRNA bovine sequence, a loop of nine non-complementary nucleotides and the reverse complementary sequence of the 19 nucleotides specific for the bovine p66Shc mRNA, which incorporates the BamH1 restriction site at the 5' end. The second oligonucleotide synthesized consists of a negative strand reverse complementary to the positive one, without the BamH1 site, but incorporating at its 5' end a HindIII restriction site (Fig. 7). The 19-nucleotide p66Shc target sequence was determined using the computer software oligoengine™ (OligoEngine, Inc., Seattle, WA) and showed no homology with any other known gene following BLAST search. The bovine sequence used for the oligo design was a segment of 336 bp obtained by PCR using primers specific for the human p66Shc sequence (Genbank number: U73377) and previously shown to specifically amplify bovine p66Shc [2]. The synthesized oligos were annealed in a concentration of 1 μg/μl in DNA Annealing solution (Ambion Inc., Austin, TX), by denaturating them at 90°C for 3 minutes, followed by an incubation step at 37°C for 1 hour. The annealed oligos were then ligated with T4 ligase (New England Biolabs, Beverly, MA) into a pSUPER plasmid (OligoEngine, Inc.), 8 bp downstream of the T7 promoter where the H1 region of the plasmid had previously been removed by HindIII and SacI enzyme digestion. The pSUPER-p66Shc hairpin RNAi plasmid was purified by QIA spin Maxiprep kit (QIAGEN Inc., Mississauga, ON, Canada) following the manufacturer's instructions and sequenced by Guelph Molecular Supercentre (Guelph, ON, Canada) to assure proper insertion of the designed oligonucleotides.

### Generation of p66Shc short hairpin shRNAi molecule by T7 *in vitro *transcription

The pSUPER-p66Shc short hairpin RNAi plasmid was linearized by HindIII digestion and the 3' overhang was converted into a blunt end by T4 polymerase (New England Biolabs). A total of 3 μg of the linearized plasmid was incubated for 2 hours at 37°C with a mixture containing: transcription buffer, 100 U of ribonuclease inhibitor, 100 mM of rNTP mix and 90 Units of T7 RNA polymerase in a final volume of 50 μl with deionized (nuclease free) water (T7 Transcription Kit, MBI Fermentas). The plasmid DNA template was digested by incubating at 37°C for 30 minutes with 3 μl of DNase I in DNase buffer (DNA-free™, Ambion Inc.), and the enzyme was inactivated by the addition of 5 μl of DNase Inactivation Reagent. The RNA product was isolated, using RNeasy^® ^Mini Kit (QIAGEN Inc.), following the manufacturer's instructions. The RNA was quantified by spectrophotometric analysis and stored at -80°C until further used. The RNA size was confirmed by running an aliquot on a 2% agarose gel together with 100 bp ladder (Invitrogen, Burlington, ON, Canada).

### Oocyte microinjection, oocyte maturation and embryo culture

Bovine ovaries were obtained from a local slaughterhouse (Better Beef Ltd., Guelph, ON, Canada) and cumulus-oocyte complexes (COCs) were collected by follicular aspiration into HEPES-buffered Ham's F-10 (Sigma-Aldrich Canada, Oakville, ON, Canada) supplemented with 2% steer serum (Cansera, Rexdale, ON, Canada), as previously described [2]. Oocytes were randomly divided into three groups: oocytes were matured, fertilized and cultured without being microinjected (control), oocytes were either microinjected with the vehicle alone (microinjection control), or with 12,000 or 24,000 molecules of p66Shc shRNAi, followed by fertilization and culture. A Narishige microinjector (Narishige, McHenry, IL) was used to microinject a volume of 0.25 picolitres of RNase-free water with or without p66Shc shRNAi molecules. The holding pipette had an outer diameter of 100 μm with an inner diameter of 30–40 μm (larger than usual to avoid stripping the cumulus cells off). The injection pipette was 4 μm in diameter and beveled at 35° (Sunpipettes-ICSI, IVFonline, Guilford, CT). The microinjector controlled the injection pipette whereas the holding pipette was controlled manually (Fig. 6). COCs awaiting microinjection were washed three times and kept in *in vitro *culture (IVC) medium, consisting of TCM-199 supplemented with 0.2 M sodium pyruvate, 0.6% penicillin-streptomycin, and 0.1% Poly Vinyl Alcohol (PVA) (Sigma-Aldrich) medium, at 38.5°C in a humidified atmosphere. Microinjection was carried out in 60 μl drops of HEPES-buffered Ham's F-10 (Sigma-Aldrich) supplemented with 2% steer serum (Cansera) under silicone oil (Paisley Products, Scarborough, ON, Canada). A total of 10–15 oocytes were individually injected at a time to reduce exposure to sub-optimal conditions. Within one hour 100–120 COCs were microinjected. Due to the relatively "harsh" microinjection procedures some oocytes ruptured or fragmented immediately following injection and were removed from the study. Triplicate groups of 80–100 of intact microinjected/non-injected oocytes per treatment group were then matured, fertilized, and the embryos cultured following the protocol previously described in detail [2]. In brief, COCs were matured in 80 μl drops of maturation medium with the addition of 0.1 μg/ml of Follicle Stimulating Hormone (FSH), 1 μg/ml of Luteinizing Hormone (LH) and 1 μg/ml of estradiol (NIH, Washington DC) under silicon oil for 22 hours at 38.5°C in a humidified atmosphere of 5% CO_2 _in air. Matured oocytes were washed as described previously [72]. Approximately 30 COCs were placed into each 100 μl drop of fertilization medium, IVF-TALP (Hepes/Sperm TALP, supplemented with 20 μg/ml heparin (Sigma-Aldrich) under oil. Frozen-thawed bovine semen (Gencor, Guelph, ON, Canada) was prepared by swim-up technique. COCs were co-incubated with sperm at a final concentration of approximately 1 × 10^6 ^motile sperm/ml for 16 to 18 hours at 38.5°C in a humidified atmosphere of 5% CO_2 _in air. Following co-incubation, the remaining cumulus cells were removed by gentle vortexing. Presumptive zygotes were transferred to 50 μl drops of IVC medium under oil. Groups of 25–30 presumptive zygotes were co-cultured with approximately 20 bovine oviductal epithelial cell vesicles for up to 9 days. To sustain development through to the blastocyst stage, 20 μl of fresh IVC medium was added to each drop after 48 hours of culture.

### Total RNA extraction

Total RNA was extracted from each pool of embryos using QIA Shredder™ and RNeasy^® ^Mini Kit (QIAGEN Inc.), according to the manufacturer's instructions. The RNA was co-precipitated using 2 μl of *See*DNA (Amersham Biosciences Corp., Baie d'Urfé, QC, Canada) as a carrier by the addition of 0.1 × volume of 3 M sodium acetate (pH 5.2) (Amersham Biosciences Corp.) and 2 × volume of ice-cold absolute ethanol. The RNA pellet was air-dried and resuspended in 8 μl of RNase-free water. Samples then underwent DNase treatment, according to the manufacturer's instructions (DNA-free™, Ambion Inc.). Following DNase treatment, samples were reverse transcribed immediately or stored at -80°C for future use.

### Reverse Transcription (RT)

RT reactions were performed on the RNA extracted from pools of oocytes/embryos using 500 ng of oligo (dT)^12–18 ^and Superscript II (Invitrogen) reverse transcriptase, according to the following protocol. The oligo (dT)_12–18 _was first added to the RNA samples and allowed to anneal by denaturing the secondary structures at 70°C for 2 min, then a mixture of 4 μl RT buffer, 1 μl of 0.1 M dithiothreitol, 1 μl of 10 mM dNTP mix, 0.5 μl of RNAsin (40 U/μl) (Promega, Madison, WI) and 1 μl of Superscript II (200 U/μl) was added and the reaction was incubated at 42°C for 1 hour, followed by a denaturing step at 70°C for 30 min. To amplify p66Shc, p53 and H2a cDNAs the primers (Table 2) that have been previously optimized for the bovine species were used [2].

**Table 2:** Oligonucleotide Primers used for Real Time amplification

Gene	GenBank accession number	Sequence	Product size (bp)	Species
p66Shc	U73377	5'-GTGAGGTCTGGGGAGAAGC-3' 5'-GGTTCGGACAAAGGATCACC-3'	336	*Homo sapiens*
H2a	U62674	5'-GTCGTGGCAAGCAAGGAG-3' 5'-GATCTCGGCCGTTAGGTACTC-3'	374	*Mus musculus*
p53	U74486	5'-CTCAGTCCTCTGCCATACTA-3' 5'-GGATCCAGGATAAGGTGAGC-3'	364	*Bos Indicus*

### Real Time RT-PCR

The PCRs were conducted in a Light Cycler apparatus (Roche Molecular Biochemicals, Laval, QC, Canada) and products were detected with SYBR Green (FastStart Master SYBR Green I mix, Roche Molecular Biochemicals). Prior to the quantification, optimization procedures were performed by running PCRs with or without the purified template to identify the melting temperatures of the primer dimers and the specific product. To measure the level of mRNA in the samples, the fluorescence values were taken at the temperature associated with the beginning of the peak for the specific product, 84°C for p53, 86°C for p66Shc and 88°C for H2a. For each quantification a 1 μl aliquot of the RT reaction was used. The standard curve was established using the DNA template in six serial dilutions ranging from 1 × 10^2 ^pg to 1 × 10^-4 ^pg. The amplification program was as follows: Preincubation for FastStart polymerase activation at 95°C for 10 minutes, followed by 40 amplification cycles of denaturation at 95°C (20°C/sec), annealing for 5 sec (20°C/sec), elongation at 72°C and acquisition of fluorescence for 5 sec (20°C/sec). After the end of the last cycle, starting the fluorescence acquisition at 72°C and taking measurements every 0.1°C until 95°C was reached generated the melting curve. Amplification was performed on the histone H2a gene as an endogenous standard to assure equal RT efficiency and for p66Shc knockdown specificity.

### Whole-mount indirect immunofluorescence and confocal microscopy of p66Shc

In order to verify the knockdown efficiency at the protein level, ~12,000 (low group) and ~24,000 (high group) shRNAi molecules were injected at the zygote stage, 18 h post co-culture with sperm or 1 hpi. Early 2–4 cell staged bovine embryos were collected and processed at 35 hpi for application of whole-mount immunofluorescence as described previously [73,74]. Sixty embryos were injected for each group over three repetitions and about 30 embryos were assessed for p66Shc immunoflurescence intensity from each repetition. Embryo pools were fixed in 2% paraformaldehyde in PBS, washed in PBS and then either processed immediately for whole-mount indirect immunofluorescence or stored at 4°C in Embryo Storage Buffer (1 × PBS + 0.9% sodium azide) for up to 3 weeks. Fixed embryos were permeabilized and blocked concurrently by room temperature incubation in Embryo Blocking Buffer (0.01% Triton X-100 + 5% Normal Donkey Serum in 1 × PBS) for 1 hour followed by one wash in fresh 1 × PBS for 30 minutes at 37°C. Embryos were incubated with primary antisera (Rabbit anti-human p66Shc; Catalog #AB3824, Millipore) at non-saturating conditions using a 1:100 dilution in Antibody Dilution/Wash Buffer (ADB: 0.005% Triton X-100 + 1% Normal Donkey Serum in 1 × PBS) at 4°C overnight. Embryos were then washed 3 times for 20–30 minutes in ADB at 37°C and incubated with FITC-conjugated secondary antibodies (Jackson ImmunoResearch Laboratories Inc., West Grove, PA, USA) at 1:200 dilution in ADB overnight at 4°C. To visualize F-actin localization and to stain nuclear DNA, the first 30 minute wash in ADB following secondary antibody incubation embryos were treated with rhodamine-conjugated phalloidin (5 μg/ml; 1:20, Sigma-Aldrich Canada Ltd., Oakville, ON, Canada) and DAPI (1 mg/ml; 1:2000, Sigma-Aldrich Canada Ltd., Oakville, ON, Canada) for 30 minutes at 37°C followed by 2 washes for 2 hours each at 37°C. Fully processed embryos were mounted onto glass slides in a drop of FluoroGuard™ Anti-Fade Reagent (BioRad Laboratories Canada Ltd., Mississauga, ON, Canada). Immunofluorescence imaging was carried out by confocal microscopy using an Olympus Fluoview Laser Scanning Confocal system on an IX81 inverted microscope equipped with a computer running the Olympus Fluoview software (v. 4.3).

### Image analysis and quantification of immunofluorescence intensity

To quantify immunofluorescence results we employed the method developed and reported by [75,76]. All microscope and image capture settings remained constant during the digital capture of confocal micrographs between embryos and between treatment groups. Acquired micrographs were saved in TIFF image format and processed using Adobe Photoshop CS2 (Adobe Systems Inc., San Jose, CA, USA), for recognition, selection and separation of the desired chromogen signal. Quantitative analysis began by separation of the green image channel representing FITC fluorescence of p66Shc protein from the red (representing Rhodamine-phalloidin-labelled F-actin) and blue (representing DAPI-stained nuclei) channels. The green channel image was converted to "Grayscale", discarding the other two color channels to ensure the signal remaining represented only FITC fluorescence. The overall luminance of each pixel in the converted image corresponded to the intensity of FITC fluorescence. Each converted micrograph was then inverted so that grey and black pixels represented areas of FITC immunofluorescence on a white background and saved as a new TIFF image file for Scion Image analysis.

The Scion Image program, Version 4.0.3.2 (Scion Corporation, Frederick, MD, USA), is a freely distributed commercial software program that mimics the performance of NIH Image program for quantification of the chromogen signal strength [75]. The greyscale inverted micrographs were opened in Scion Image and following the methods of [75], the mean density of the chromogen signal strength (SS) of each image was measured and recorded. The average SS from at least three micrographs representing no primary antibody controls was subtracted from the SS values of the measured images from each treatment group to produce an adjusted relative SS value. Relative signal strength (RSS) values represented in pixels were plotted on the graph.

### Assessment of cleavage, blastocyst and arrest frequencies

Developmental potential was assessed by evaluating the frequency of embryos cleaving at day-2 post insemination (p.i.), and percentage of those reaching the blastocyst stage at day 8–9 p.i. Embryos were considered "arrested" when still appearing as "morphologically normal" 2–4 cell embryos at day 8 post insemination (Fig. 1). The frequencies of blastocyst formation and embryo arrest were calculated as a proportion of the total number of cleaved embryos. The proportion of spontaneously cleaved oocytes was evaluated in each IVF experiment using a group of oocytes (10 to 15 oocytes per experiment) matured and cultured, omitting the fertilization step. The spontaneous cleavage rate was calculated at 35 hpi as the number of oocytes cleaving in absence of sperm over the total number of oocytes in each group.

### Statistical analysis

Statistical analysis was performed using one-way ANOVA and Fisher LSD multiple comparison test. The non-parametrical equivalent test (Kruskal-Wallis) was used when samples did not meet the assumption of normal distribution or homogeneity of variance. The results are presented as the means ± SEM from three independent experiments. Data were analyzed by MINITAB™ software or SPSS^®^, Version 14.0 (SPSS Inc., Chicago, IL, USA) and means were considered significantly different when P < 0.05 using the least-significant-difference test.

## List of abbreviations

RNAi – RNA interference, shRNAi – short hairpin RNAi, IVF – in vitro fertilized, IVP – in vitro produced, ROS – reactive oxygen species, hpi – hours post-insemination, GV – germinal vesicle, dsRNA – double stranded RNA

## Authors' contributions

LAF participated in the design of the study, performed the experiments including construction of the p66Shc RNAi molecule, Real Time PCR analyses, embryo development analyses and statistical analyses, and drafted the manuscript. PM carried out the zygote microinjections and semi-quantitative immunofluorescence analyses of p66Shc protein levels. GFM performed the oocyte microinjections and helped to draft the manuscript. ES carried out the oocyte retrieval and in vitro embryo production. WAK contributed to the evaluation of the data and the manuscript contents, and acquired funding for the project. DHB coordinated the study, provided critical review of the results and manuscript contents, revised the manuscript and acquired the funding for the research program. All authors read and approved the final manuscript.
